# Are heterotrophic and silica-rich eukaryotic microbes an important part of the lichen symbiosis?

**DOI:** 10.1080/21501203.2014.974084

**Published:** 2014-10-28

**Authors:** David M. Wilkinson, Angela L. Creevy, Chiamaka L. Kalu, David W. Schwartzman

**Affiliations:** a School of Natural Science and Psychology, Liverpool John Moores University, Byrom Street, LiverpoolL3 3AF, UK; b Environmental Research Institute, University of the Highlands and Islands, Ormlie Road, Thurso, Caithness, KW14 7EE, UK; c Department of Biology, Howard University, Washington, DC20059, USA

**Keywords:** lichens, protists, symbiosis, testate amoebae, diatom, chrysophyte

## Abstract

We speculate that heterotrophic and/or silica-rich eukaryotic microorganisms maybe an important part of the lichen symbiosis. None of the very few studies of heterotrophic protists associated with lichens have considered the possibility that they may be of functional significance in the lichen symbiosis. Here we start to develop, currently speculative, theoretical ideas about their potential significance. For example, all the protist taxa identified in lichens we sampled in Ohio USA depend on silica for growth and construction of their cell walls, this could suggest that silica-rich lichen symbionts may be significant in the biogeochemistry of the lichen symbiosis. We also present arguments suggesting a role for protists in nitrogen cycling within lichen thalli and a potential role in controlling bacterial populations associated with lichens. In this necessarily speculative paper we highlight areas for future research and how newer technologies may be useful for understanding the full suite of organisms involved in the lichen symbiosis.

## Introduction

The idea that lichens were a composite organism, formed of both fungi and algae in some sense working together, was highly controversial when proposed in the mid-nineteenth century. It took decades before this idea started to establish itself as the orthodox position – although the detailed nature of the relationship remained controversial (Sapp 1994). During the first half of the twentieth century various authors suggested that a wide range of other microorganisms may also be involved in the lichen symbiosis. However, these ideas were not widely believed, for example in a mid-century review of lichen biology Brightman (1959) suggested that these results were ‘unlikely… to be very near the truth’. With the rise of molecular approaches to such questions these ideas have again become areas of current research and there is renewed interest in the role bacteria may play in the lichen symbiosis (e.g. Grube and Berg (2009); Bates et al. (2011); Mushegian et al. (2011); Cardinale et al. (2012)). Most of this recent work has focused on prokaryotes and much less attention has been given to eukaryotic microbes potentially associated with lichens (but see Bates et al. (2012) and Anderson (2014) – discussed below). Here we raise a number of potentially important questions about the role of various eukaryotic microbes, illustrated by some of our own previously unpublished observations, and conclude by suggesting ideas for further work that should help clarify our, currently speculative, ideas on the potential role of a wide range of protists in the lichen symbiosis.

## Non-molecular data on protists associated with lichens

To look for protists associated with lichens we collected 52 lichen samples during 2008–2010 from the Chesapeake & Ohio Canal National Historical Park, Maryland, USA (the study Kalu 2010 provides more extensive details). Specimens of the lichen *Flavoparmelia caperata* were collected from bark and *F. baltimorensis* from rock surfaces. Protists were cultured in a media made following the recipe of Aoki et al. (2007) which gives a culture solution with a silica concentration of 120 ppm.

Two protists were isolated from our cultures, the chrysophyte *Ochromonas crenata* and the testate amoebae *Corythion dubium. Ochromonas* was found from all lichens in our samples throughout the year; to the best of our knowledge this is the first report of a chrysophyte in a lichen. *Corythion dubium* was rarer and only cultured from lichens (of both species) collected in December 2009. Both of these taxa are likely to feed on bacteria for at least part of their diet, the genus *Ochromonas* contains many mixotrophic species (Margulis and Chapman 2009) and *C. dubium* has been recorded feeding on bacteria and protists such as flagellates (Wilkinson and Mitchell 2010). Culture-based studies cannot quantify the abundance of the different protists in the lichen and will only identify those taxa suited to the culture conditions. Since both observed protist taxa utilise silica in constructing their cell walls (*O. crenata* forming silica-rich stomatocysts*)*, they will have been favoured by our culture media. In addition, culture-based studies cannot say much about the location of the protists in the original lichen sample – for instance, are they surface living or found inside the thallus? To address these issues some of the samples were examined directly by light and scanning electron microscopy (SEM). Light microscopy of fragments of shredded lichen thallus additionally confirmed the presence of two more testate taxa; *Euglypha rotunda* and a rather degraded test of *Assulina sp*. We attempted to establish the *in situ* presence of testates within the thallus by SEM examination of fractured thallus fragments – but were unsuccessful in finding any testates within the lichen using this method. Our experience of working on these samples illustrates the difficulty of investigating such questions using conventional culture techniques and/or direct microscopy.

**Figure 1.**
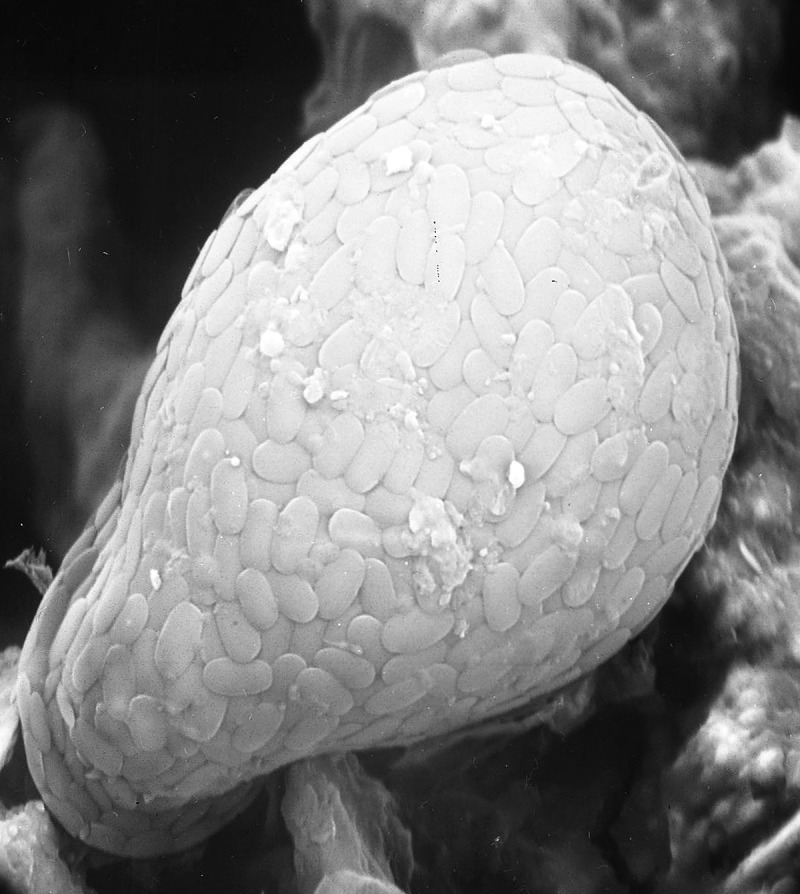
*Corythion dubium* from the underside of the lichen *Flavoparmelia baltimorensis* collected from near the Cheapstake and Ohio canal (close to the site of the lichens used in the culture studies described in this paper) – this specimen was erroneously described as a ‘chrysophyte’ in Schwartzman (1999). The test of this taxon varies between approximately 25 and 65 μm in length (Ogden and Headley 1980). In addition to testate amoebae diatoms were also found on the underside of this lichen thallus (an example is illustrated on Schwartzman 1999, p. 70).

Testate amoebae have been recorded as associated with lichens in a range of previous studies. For example one of us (David W. Schwartzman) had previously recorded both *C. dubium* and diatoms from the underside of the lichen *Flavoparmelia baltimorensis* (Figure 1). In addition, Anderson (2014) has recently recovered a range of heterotrophic nanoflagellates and naked amoebae from lichens growing on trees using an approach similar to our lichen ‘shredding’ method, while Roberts and Zimmer (1990) found a range of protozoa (mainly ciliates) in a culture-based study of lichens in Northern Ireland. Lichens can be the dominant ‘vegetation’ in many inhospitable habitats such as high mountains, polar areas and hot deserts (Stephenson 2010) and although their fossil record is extremely poor it is often assumed that they may have formed some of the first terrestrial ‘vegetation’ (e.g. Lenton and Watson 2011). There are a small number of studies showing testate amoebae species associated with lichens in the Arctic (Beyens et al. 1986, 1990). Bamforth (2008) recorded not only testates from lichens in the Utah Desert, USA, but also naked amoebae and ciliates too. One of the reasons that lichens survive in such extreme habitats is that they can have a remarkable ability to tolerate complete drying out for long periods (Gilbert 2000). Any protist living in or on them must be able to do the same. We speculate that this may favour testate amoebae which are able to encyst inside their shells when conditions are not favourable. These studies confirm the presence of various protists living in or on lichens but raise many new questions.

## Are protists associated with lichens of any functional importance?

If – as now seems possible – a wide range of bacteria living in and on the lichen thallus plays an important role in the function of the lichen symbiosis (Grube and Berg 2009), then the presence of potential bacterial predators is obviously of some interest. Currently eukaryotic microbes in lichens tend to be viewed as just inhabiting the lichen thallus rather than being an integral part of the lichen system (e.g. Bates et al. 2012). However in soils, protists such as testate amoebae have a role as predators – and hence contribute to nutrient cycling in soils (Wilkinson and Mitchell 2010). There is clearly a possibility that similar interactions may be occurring within some lichen thalli.

For one potential example consider our empirical results outlined earlier. Our silica-rich culture media isolated two taxa whose growth is dependent on supplies of available silica. The lichen samples were collected from two different substrate types, so silica may have come from different environmental sources to these lichens. Silica from silicate rock substrates (such as the quartzites and schists) exposed in our study site is likely solubilised by chemical weathering facilitated by lichen activity (Schwartzman 1999). Furthermore, rain water commonly contains small silicate mineral dust particles, as well as soluble silica, which could be a source of silica for the lichen symbionts. In addition, silica could transfer into *F. caperata*, from the bark lichen substrate. The observation of these silica-rich lichen symbionts suggests they may be fairly common and so potentially significant in the biogeochemistry of silica in the lichen symbiosis (Schwartzman 1999). For example, they may act as stores for soluble silica and these non-obligate symbionts may facilitate the availability of other nutrient elements to the obligate lichen symbionts. Similar ideas about the role of testate amoebae with silica-rich shells have been suggested for soils (Aoki et al. 2007). One way of extending such work would be to compare the protists associated with lichens from a range of silica-rich and silica-poor substrates.

Additionally heterotrophic protists may have important functions in other nutrient cycling processes within lichens. For example Ellis et al. (2005) showed recycling of nitrogen from older lichen tissue to more actively growing parts of the thallus – however, they were unsure of the mechanism for nitrogen movement. More recently, Cardinale et al. (2012) showed higher bacterial levels in older parts of lichen thalli and suggested that these may play a role in such nitrogen recycling. Clearly protists feeding on bacteria are of some potential interest in this context. In addition, Anderson (2014) showed a positive correlation between bacterial numbers and heterotrophic protist numbers in lichen thalli. There are obviously interesting questions about what controls bacterial populations in and on lichens (these questions apply irrespective of the functional significance of these bacteria). Could heterotrophic protists perform an immune system–like role – helping to control bacterial populations?

Currently, our rather limited understanding of protists (other than green algae) in lichens prevents us from determining whether they have any functional significance. Clearly key starting questions are how diverse are these taxa in lichens and what is their abundance? A long list of taxa may be of little ecological relevance to the lichen if they are always rare. In addition, it is important to know to what extent they are confined to surface biofilms or if they are distributed throughout the thallus. Our own experience suggests that conventional microscopic approaches (either light or SEM) fail to adequately determine the distribution of protists in the lichen thallus, and molecular approaches may be more useful. Bates et al. (2012) used molecular approaches to characterise eukaryotes within the thalli of *surface sterilised lichens.* They identified a long list of eukaryotic taxa but were unable to say anything about their abundance – as pointed out above this is crucial ecological information.

The results from our investigations raise many questions about the potential links between heterotrophic protists, lichens, their associated bacteria and biogeochemical cycling. *Indeed the key aim of this paper was to raise new questions and outline potential approaches to addressing them.* Based on the limited data so far available the role of protists in the lichen symbiosis is an open – and potentially fascinating – question.
